# Algorithms for a Single Hormone Closed-Loop Artificial Pancreas: Challenges Pertinent to Chemical Process Operations and Control

**DOI:** 10.3390/pr4040039

**Published:** 2016-10-18

**Authors:** B. Wayne Bequette, Faye Cameron, Nihat Baysal, Daniel P. Howsmon, Bruce A. Buckingham, David M. Maahs, Carol J. Levy

**Affiliations:** 1 Department of Chemical and Biological Engineering, Rensselaer Polytechnic Institute, 110 Eighth St., Troy, NY 12180-3590, USA; 2 Stanford University, 780 Welch Road, CJ320H MC 5776, Palo Alto, CA 94304, USA; 3 Barbara Davis Center for Diabetes, University of Colorado, Denver, 1775 Aurora Court, Aurora, CO 80045, USA; 4 Icahn School of Medicine at Mt. Sinai, 1 Gustave A. Levy Place, New York, NY 10029, USA

**Keywords:** artificial pancreas, glucose control, type 1 diabetes

## Abstract

The development of a closed-loop artificial pancreas to regulate the blood glucose concentration of individuals with type 1 diabetes has been a focused area of research for over 50 years, with rapid progress during the past decade. The daily control challenges faced by someone with type 1 diabetes include asymmetric objectives and risks, and one-sided manipulated input action with frequent relatively fast disturbances. The major automation steps toward a closed-loop artificial pancreas include (i) monitoring and overnight alarms for hypoglycemia (low blood glucose); (ii) overnight low glucose suspend (LGS) systems to prevent hypoglycemia; and (iii) fully closed-loop systems that adjust insulin (and perhaps glucagon) to maintain desired blood glucose levels day and night. We focus on the steps that we used to develop and test a probabilistic, risk-based, model predictive control strategy for a fully closed-loop artificial pancreas. We complete the paper by discussing ramifications of lessons learned for chemical process systems applications.

## 1. Background

The human body uses feedback control to regulate many physiological variables such as body temperature, blood pressure, and cardiac output, improving the consistency and efficiency of other bodily functions. A prime example is the regulation of the concentration of glucose in the blood by the beta and alpha cells of the pancreas. If the blood glucose concentration is high, the beta cells produce more insulin, allowing glucose to diffuse into tissue cells and the liver where it is stored as glycogen. When the blood glucose concentration is low, the alpha cells produce glucagon, which converts the glycogen from the liver back into glucose, thus raising the blood glucose concentration. The body’s regulation of the blood glucose concentration is disrupted by diabetes mellitus, which occurs primarily in two forms: type 1 (T1D) and type 2 (T2D). Type 2 diabetes is the most common form, representing 90%–95% of diabetes cases. In T2D the beta cells continue to produce insulin but insulin resistance in the body weakens its ability to respond quickly to meal disturbances, causing more sustained elevated glucose levels and with them chronic negative health consequences. In T1D, the body rejects the beta cells and so the body’s insulin production.

Before the 1922 discovery of insulin by Banting and Best [1], a diagnosis of T1D was a death sentence. During the first several decades after the discovery of insulin, limitations in insulin pharmacodynamics and the lack of glucose testing made it extremely important for individuals with T1D to be consistent in their daily activities, meal consumption and insulin delivery. Glucose testing technology has improved from the urine testing in the 1950s which required the mixing of reagents to at-home self-monitoring blood glucose (SMBG) meters in the 1980s and finally to continuous glucose monitoring (CGM) in 2006 [2].

Similarly, insulin has improved through more stable formulations and both rapid and long-acting formulations. However, even with the state-of-the-art technology, and a well-calibrated system, patients must calibrate their CGM at least twice per day and determine an appropriate dose of insulin to inject at least four times per day in order to reach the American Diabetes Association’s goal of HbA1c < 7.0% [3]. A closed-loop artificial pancreas uses the real-time measurements provided by a continuous glucose monitor to provide automatic insulin delivery through an insulin pump. Additional details of the artificial pancreas are explored below.

### 1.1. Insulin

Currently, insulin can be broadly categorized as either long-acting or rapid acting. Someone on multiple daily injection treatment will typically inject long-acting insulin once per day to take care of basal (steady-state) needs. Then, they will inject rapid-acting insulin to compensate for meals or snacks, or as a correction bolus if their blood glucose is high. For an overview and history of insulin, see Hirsch [4]. It is worth noting that insulin pharmacodynamic action, the effect of insulin on the glucose level vs. time, is obtained through feedback control experiments, though usually implemented manually; a bolus of insulin is injected subcutaneously and glucose is administered intravenously (i.v.) to maintain a desired blood glucose concentration and the resulting i.v. glucose infusion rate is the insulin action profile [5–7]. There is much inter- and intra-subject variability, so pharmacodynamics profiles are usually averaged over 10 or more experimental subjects.

### 1.2. Components

A modern closed-loop artificial pancreas requires three major components: a continuous glucose monitor (sensor, CGM), an insulin pump, and a controller to command the insulin pump based on the data from the CGM. The first fully closed-loop device, known as the Biostator, sampled the blood intravenously to analyze glucose and infused insulin directly into the bloodstream [8]. This large-scale bedside device was not portable, and the direct, intravenous sampling of blood and infusion of insulin led to unacceptable risks in conceivable portable applications at that time. Thus, the focus over the past few decades has been on the development of glucose sensors that sample the interstitial fluid just under the skin, and insulin pumps that deliver insulin subcutaneously. While the state of continuous insulin infusion pumps is advanced, with many devices available for over 20 years, only recently has continuous glucose sensing advanced to the state where they can be reliably used as part of a fully closed-loop artificial pancreas (see [9,10] for reviews of CGM technology).

### 1.3. Challenges to Closed-Loop Control

At first glance an experienced control engineer may think that developing a closed-loop artificial pancreas is a simple single input, single output (SISO) engineering problem, involving the measurement and control of blood glucose by manipulating the insulin infusion rate. A concise listing of the challenges follows.

Physiological Challenges:

- The short-term risk is much higher for low than for high blood glucose concentrations
- A person’s insulin sensitivity (“process gain” relating insulin to glucose) often varies throughout the day, and significantly over longer time scales due to illness or significant changes in exercise regimen
- The pharmacokinetics (concentration) and pharmacodynamics (effect) of subcutaneously delivered insulin are much longer than that for insulin delivered intravenously. Even rapid-acting insulin is “slow” relative to meals with a peak effect time of around 75 min.
- Different types of meals, even with the same number of carbohydrates, can have different time-scale effects on blood glucose
- Exercise, usually aerobic, can rapidly bring glucose values down; however a period of anaerobic exercise can cause glucose levels to rise
- Meal disturbances have a one-sided effect and require large amounts of insulin relative to the normal based (steady-state needs). For example, a typical basal (steady-state) infusion rate may be 1 U/h, yet a large meal could require that 15 U be administered during a 5 min sample interval
- While the steady-state insulin can be withheld when using an insulin pump, insulin administered cannot be removed.

Component Challenges:

- Unreliability in responding to alarms and failure to provide desirable information such as the carbohydrates in a meal
- Pump insulin infusion sets can fail and need to be replaced every three days (or sooner)
- Sensor (continuous glucose monitor, CGM) signals lag the capillary blood glucose by around 5–10 min and there are typically other sensor-related delays (numerical filtering, for example) of another 5–10 min
- Current CGMs need to be calibrated twice daily, using reference self monitoring blood glucose (SMBG) “fingerstick” measurements; these reference measurements are uncertain due to strip manufacturing limits and patient error (not washing and drying hands thoroughly, for example)
- CGMs are currently approved for up to seven days of use, although patients will often go 14 or more days on a single sensor
- CGM signals can have artifacts, such as pressure induced sensor attenuations (PISA) due to individuals rolling over on their sensor during sleep
- Bluetooth signals can drop-out or the devices sending and receiving (CGM receiver to smart phone) can lose connectivity.

Many of these challenges are addressed briefly in this paper.

### 1.4. Safety

Similar to chemical process plants and aircraft, safety must be an overriding concern with a closed-loop artificial pancreas [11]. In the short-term the greatest risk is due to hypoglycemia, which occurs largely due to over-delivery of insulin or to exercise. Hyperglycemia is a longer-term risk and is largely due to under-delivery of insulin, which can occur because of poor control, reduced insulin sensitivity, or infusion set faults. It is common to replace infusion sets at 3-day intervals, but sets can fail before then.

## 2. Control Algorithms

The Juvenile Diabetes Research Foundation (JDRF) proposed a roadmap or series of natural steps in the development of a closed-loop artificial pancreas, from a very low glucose suspend algorithm that simply shuts off the insulin pump when glucose is too low (an on-off algorithm) and focused primarily on overnight, to a dual hormone strategy that manipulates both insulin and glucagon and functions day and night, handles meals and exercise, etc. [12]. While we have been involved in the development of a predictive low glucose suspend (PLGS) system that has been tested in over 7000 subject-nights of at-home use [13,14], we will focus our algorithms discussion on fully closed-loop systems meant to operate 24/7 and manipulate only insulin. For a direct comparison of single and dual hormone results see Haidar et al. [15], and for more general overviews of challenges and algorithms involved in the development of a fully closed-loop artificial pancreas, see [16–21].

### 2.1. Algorithms Overview

Much like the debates about the gap between theory and practice of advanced control that occurred at chemical process control conferences through the 1990s (see Shinskey [22], for example), there has been a similar debate about the use of PID and MPC algorithms for a closed-loop artificial pancreas. As an example, Bequette [23] assessed the merits of MPC, while Steil [24] reviewed the advantages of PID. Cameron et al. [25] compared MPC, PID, and enhanced MPC with an “optimal”, but not realizable basal-bolus strategy in a simulation-based study. Recently, Pinkster et al. [26] reported clinical trial results of a head-to-head comparison of MPC and PID, and concluded that MPC had better performance.

### 2.2. Multiple Model Probabilistic Predictive Control (MMPPC)

We have had success with various iterations of a strategy that we call multiple model probabilistic predictive control (MMPPC). The objectives of MMPPC are to reduce the burden on the patient by:

- Not requiring meal announcement
- Mitigating low glucose concentrations due to exercise
- Taking advantage of knowledge of typical eating and sleeping patterns
- Taking advantage of announced sleep
- Targeting as low a glucose concentration as is safe.

The first version of our algorithm was developed as part of a PhD dissertation [27] and focused on anticipating and detecting meals as well as dosing insulin based on the asymmetric risk of hypo- vs. hyperglycemia, using a risk measure similar that that developed by Kovatchev et al. [28]. Another novel feature of the algorithm, initially called extended model predictive control (EMPC), includes uncertainty in the future predictions of blood glucose, especially around meals. Thus, insulin was dosed to minimize a probability-weighted risk index [25]. Finally, common sleep and meal patterns were incorporated from national survey data [29–31].

One of the major challenges in developing a fully closed-loop artificial pancreas is that subjects will neglect to provide a meal-related insulin bolus (feedforward control), which often results in a period of post-meal hyperglycemia (high blood glucose) [32]. Our MMPPC approach anticipates and then detects when a meal is being consumed, estimates the meal size, and begins to more aggressively inject more insulin. Our approach includes the knowledge of typical eating patterns; for example, if someone has just consumed a large meal they are unlikely to eat again for several hours. At each time step the controller delivers insulin to assure a low probability that an individual will become hypoglycemic (low blood glucose). Similarly, when a subject is likely to be sleeping (which can be inferred from a 3-axis accelerometer), the insulin delivery is less aggressive, but again targeting a low probability of hypoglycemia. The algorithm also accounts for the increased insulin sensitivity and reduced need for insulin during exercise.

### 2.3. Fault Detection

Our core algorithm assumes that the hardware components are working correctly. When they fail, glucose regulation and patient safety can be compromised. Two major faults are losses in infusion set actuation (LISAs) and pressure-induced sensor anomalies (PISAs), as discussed below [33].

Insulin infusion sets are prone to a variety of faults. The primary mitigation of these faults is the widely accepted recommendation that insulin infusion sets are changed every three days. Since there is significant variability in the lifespan of these sets [34] many infusion sets are replaced before they need to be, driving up costs and increasing patient burden. Modern infusion pumps can monitor the back-pressure on an infusion catheter, but many LISA faults do not trigger these alarms. Consequently there is some research on using analytical redundancy to detect LISAs through their effect on the control of the glucose level. Since detection of these faults results in the same patient action (i.e., replace the infusion set in a new patch of skin), most research on LISAs has been restricted to the fault detection problem rather than the fault identification problem.

Until recently (July 2016) continuous glucose monitors were only approved for adjunctive therapy and insulin dosing strategies could not be prescribed based solely on readings from these devices. However, modern glucose sensors coupled with frequent calibration and long sensor lifespans increase sensor accuracy and reliability.

While CGMs are accurate enough for closed-loop control, they are prone to excessive inaccuracy when excess pressure is applied to the sensor for 10 or more minutes, causing an exponential decline in the CGM readings and later a steep rise to accurate readings when the pressure is removed. These PISA events are problematic for artificial pancreas devices since they can cause control algorithms to reduce or cease insulin administration, resulting in increased blood glucose concentrations. On the other side, the steep rise in glucose levels could be interpreted as a meal and lead to excessive insulin and hypoglycemia.

There are several rules/constraints to be checked during real-time CGM operation in order to understand if the reading is healthy or faulty. Generally, a PISA event starts with a sudden decrease in glucose levels violating physiological rate-of change limits. On the other end a PISA event generally ends at least 15 min later and with a negative rate of rate of change. A more detailed explanation of the PISA detection algorithm has been given by Baysal et al. [35]. In this methodology, a Kalman filter algorithm was used to provide glucose predictions at 1-min intervals, which are then updated when a new CGM reading is available (typically at 5-min intervals). It was shown that 88.34% of the PISA events during 1125 nights of outpatient trial were successfully detected by using this technique.

## 3. Clinical Trial Process

The need for, and importance of, human clinical trials makes the development of a closed-loop artificial pancreas substantially different from the majority of chemical process control projects. Regulation by the Federal Food and Drug Administration (FDA) makes the process perhaps more similar to Nuclear Power Plants or the development of a new pharmaceutical.

While simulators and animal models exist for T1D (e.g., [36]), they do not fully mimic the variability observed in humans, thus testing algorithms in closed-loop with human subjects is critical. To conduct clinical trials a number of regulatory-related steps are involved, including Investigational Device Exemption (IDE) applications to the US Food and Drug Administration (FDA), oversight by Institutional Review Boards (IRB), reports to and from Data and Safety Monitoring Boards (DSMB), and clinical trial registration. These steps alone can easily delay a project by a month or more.

The clinical protocol, while similar to experimental or operational procedures for laboratory experiments or process operations, is very specific. Since it is part of the regulatory packet, violations of the protocol need to be reported to the IRB and other groups. The protocol contains details such as how the subjects will be enrolled and what visits are required before the actual clinical study begins, the time/location of the study, the requirements that need to be met (blood glucose within a range, no blood ketones) before closed-loop can begin. The protocol clearly states the specific inclusion or exclusion criteria for patients participating in the study—this may involve age ranges, total daily insulin dose (scaled by subject weight), daily carbohydrate intake, Hemoglobin HbA1c (an indicator of how well the subject controls their glucose levels) and health status.

Artificial pancreas related clinical trials have used a variety of closed-loop performance metrics based on CGM (and direct blood glucose measurements, particularly in the inpatient studies) values, including mean, median, and % time spent in specific ranges. Unfortunately, the metrics have not been consistent, with different ranges, etc., reported in different studies. The primary goal of the article by Maahs et al. [37] was to develop consensus metrics for clinical trial results reporting. Fundamentally these measures must reflect how well the glucose levels were controlled, mean and distribution, as well as how much help the controller received by way of human intervention. The human intervention is almost exclusively in the form of extra carbohydrates to compensate for excess insulin. Other considerations related to hardware performance include the amount of time that valid sensor signals were received, the amount of time spent in closed-loop, and rate of infusion set or sensor failures.

To assist sponsors in putting together IDE and Premarket Approval (PMA) applications, the FDA issued a guidance document in 2012 [38].

## 4. Clinical Trial Results

While over 87 clinical trials have been published related to the closed-loop artificial pancreas, we focus exclusively on our results. For a broad overview, see the clinical trial database maintained by the Doyle group: http://thedoylegroup.org/apdatabase [39].

**Figure 1:**
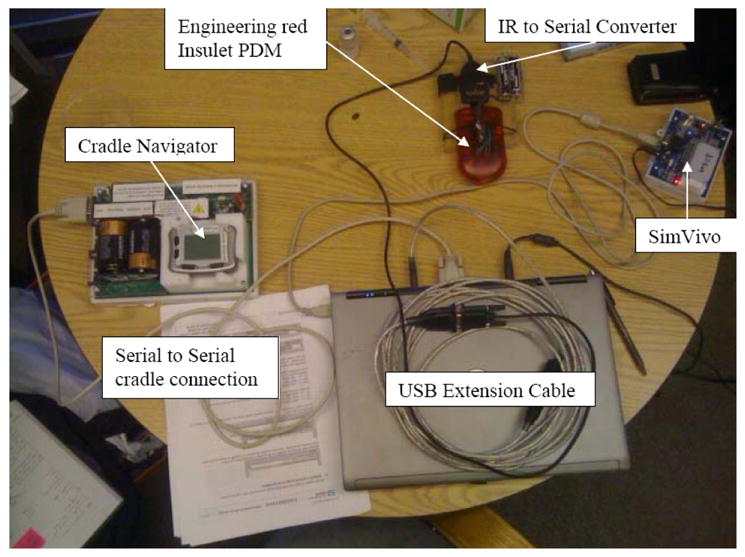
Components used in the inpatient studies conducted in Cameron et al. [41].

Our trials began with simulation studies using FDA accepted simulator. However, the simulator was only approved on the population level and we were almost by definition most interested in the extreme patients where our controller failed. This made the simulator useful in a regulatory context, but not particularly useful in fine-tuning any parameter settings. From there we have conducted clinical trials on more and more sophisticated hardware and in more and more independent living environments. Our early efforts involved the installation of our MMPPC algorithm on a laptop-based artificial pancreas system (APS) [40] used overnight in a hospital (inpatient) environment. An Abbott Navigator CGM, and an Insulet Omnipod insulin pump were used in the device shown in Figure 1. The overnight studies (32 h) involved five unannounced meals and 10 subjects [41].

The clinical settings also restricted patients. For the inpatient studies patient’s blood glucose level was tested intravenously every hour. This seriously restricted what they could do and where we could take them. Add to that the central fixture in any hospital room is the bed, and you get decidedly sedentary behavior. With hotels we get more realistic behavior, but avoid a lot of the variability of real world living. Patients were woken up in the morning and warned of the late hour. This lack of variability makes real-time detection of wake and sleep seem less important.

**Figure 2:**
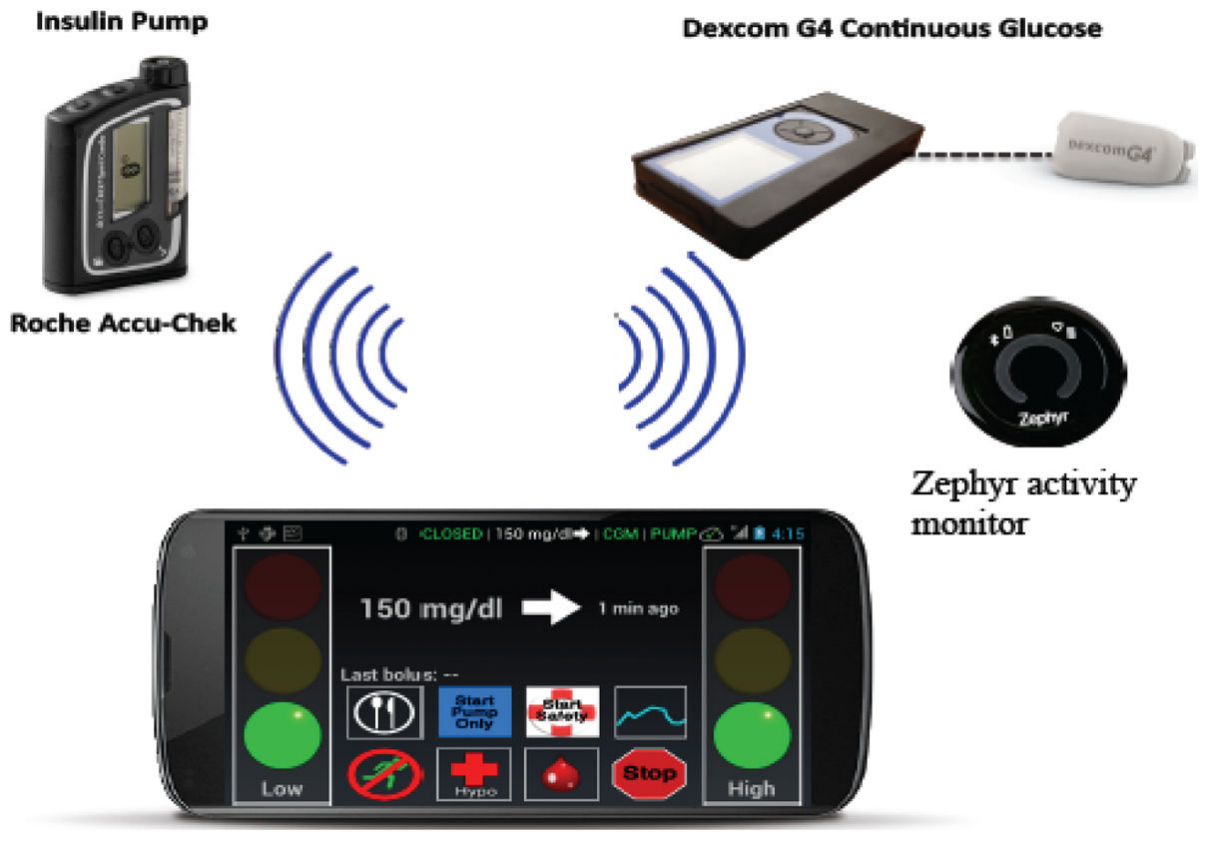
Portable system used in more recent studies reported in Cameron et al. [43,44].

Since the laptop-based system was clearly not portable, we partnered with colleagues from the University of Virginia, who had developed the DiAs (Diabetes Assistant) platform specifically for ambulatory-based clinical trials [42]. This portable platform, shown in Figure 2, was used in our more recent inpatient and hotel studies [43,44].

**Figure 3:**
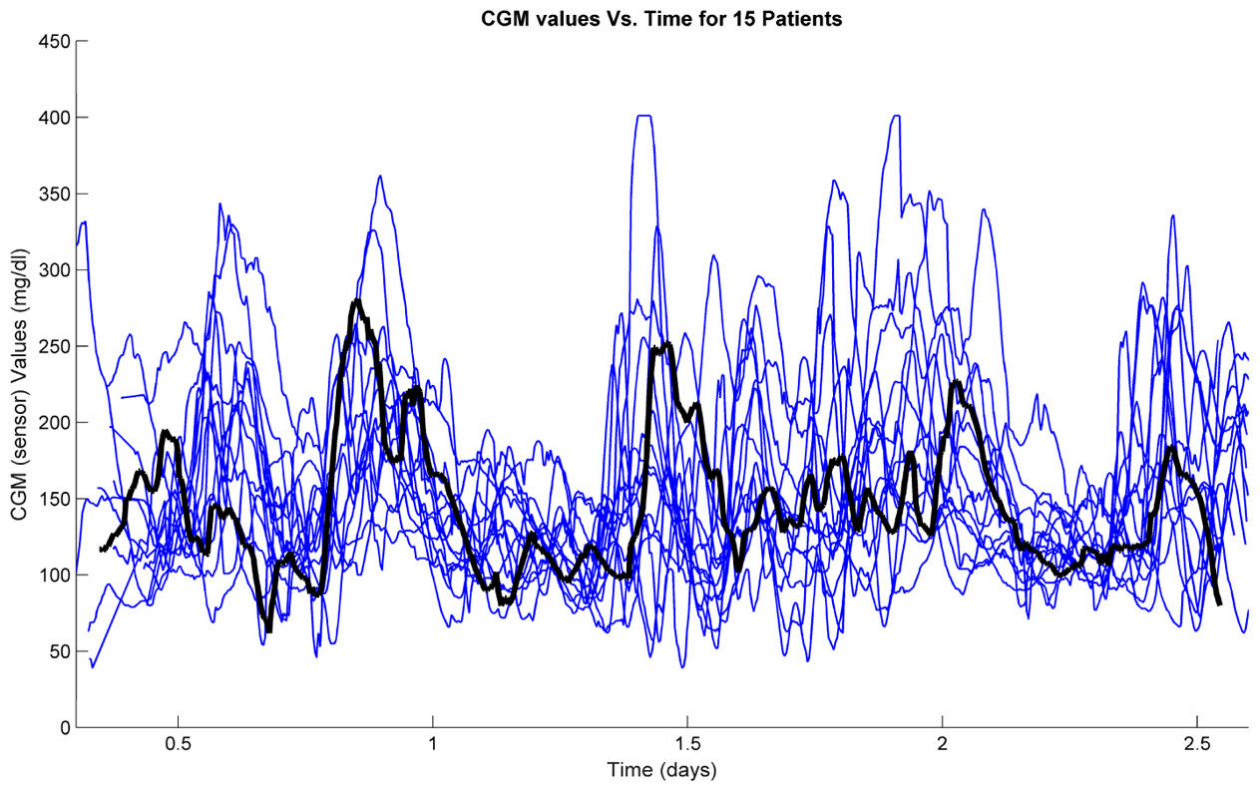
Continuous glucose monitoring (CGM) traces from the hotel study involving 15 subjects, reported by Cameron et al. [44].

Even with all of this consistency, the trial results have a lot of variability. Figure 3 shows the results across 15 patients in hotel studies at three clinical sites, with one of them highlighted. All 15 patients had roughly synchronized exercise intensity and duration, meal times and amounts, and sleep/wake periods. Part of the variability is that we had variable initial conditions; the sole starting requirements were that patients were required to have glucose concentrations between 80 and 250 mg/dL, and have a significant amount of insulin in the last 4 h. The variability comes in part from the individual person’s physiology, their stress level, the specific food they food, the order in which they ate their food, whether they had a bedtime snack, the intensity of exercise [45], how fit they are, and lastly, their history of low glucose levels.

For the highlighted patient, they ate and were given insulin just before the study and then at 13:30 (0.56) days had a very fast meal response that led to too much insulin. That extra insulin and exercise at 15:00 (0.64 days) led to low glucose concentrations and a hypoglycemic intervention (extra carbohydrates to raise the glucose concentration). By comparison, the exercise the next day at 15:00 (1.6 days) had minimal effect despite a similar amount of on board insulin. Also, the remaining meals including the one at 23:30 (0.94 days) did not lead to excessive insulin. Indeed, there was only one hypoglycemic intervention. The remaining glucose levels were, for a patient not announcing meals, quite well controlled.

## 5. Generalization to Process Systems Engineering

### 5.1. Generalization to Chemical Process Operations

What have we learned that can be generalized and applied to chemical process operations and control? The most direct application is to batch and semi-batch processes, where the dynamic behavior and objectives change during the course of the batch. Individuals have schedules/activities that change from day-to-day, imposing challenges that are similar to multi-product chemical plants. The major concern with hypoglycemia is analogous to chemical product purity and related specifications, where there can be severe economic penalties when these specifications are violated. The long time-scale pharmacodynamic action of insulin makes it important to keep track of “insulin on board” (IOB), since insulin injected two hours ago continues to have an effect on glucose for the next three hours or so [7]; this is most analogous to an exothermic semi-batch reactor, where overfeeding can cause excess reactant to accumulate, possibly resulting in a large exothermic temperature excursion when cooling temperature limitations are encountered [46].

Continuous glucose monitors used for the feedback signal create what is essentially an inferential control (state estimation) problem, since at least two reference glucose (fingerstick) measurements per day are required to keep the sensor in calibration; this is analogous to the combined use of continuous process measurements along with infrequent product composition measurements that have some level of uncertainty.

### 5.2. Generalization to Process Development

There are also process development analogies—for example, initial clinical trials are conducted in a hospital environment (inpatient) on a limited number of subjects, analogous to laboratory scale chemical process development. Like process development, lessons learned and data collected during the initial inpatient studies allow model development and a tailoring of the protocols for the next clinical trial stages. Similar to pilot plant testing, where multiple scale reactors are studied, hotel and camp studies involve “scale-up” where many subjects, with different characteristics and behaviors, can be studied simultaneously as a transition between R & D and use at-home. Initial at-home studies in a sense represent the final pilot plant scale, with shorter-term (two week) studies followed by much larger trials of up to six months. A number of artificial pancreas groups throughout the world are now at the larger clinical trial stage, which will be followed by extensive trials for commercially available devices.

## 6. Conclusions

The development of a closed-loop artificial pancreas involves many individuals with a wide variety of expertise. While much effort is spent on algorithm development, an order of magnitude more effort is required to develop platform(s) for closed-loop studies, perform clinical studies in clinical research centers, hotels/camps, and outpatient (at home). The process is similar to process development, scale-up for pilot plant studies, followed by full scale manufacturing. There are many more oversight and regulatory bodies involved in the development of a biomedical device, such as a closed-loop artificial pancreas, including Institute Review Boards (IRB), Data Safety Monitoring Boards (DSMB), FDA Investigational Device Exemption (IDE) application and review, and funding agencies (such as the NIDDK). Frequent follow-up and annual reports are required for each of these groups.
